# Evaluation of the effect of the capitation compensation mechanism among pulmonary tuberculosis patients with a full period of treatment

**DOI:** 10.1186/s40249-021-00861-0

**Published:** 2021-05-25

**Authors:** Xing-Yu Hu, Guang-Ying Gao

**Affiliations:** School of Public Health, Capital Medical University, No.10 Xitoutiao, Youanmenwai Street, Fengtai District, Beijing, 100069 China

**Keywords:** Pulmonary tuberculosis, Payment method, Capitation, Bundle payment, Evaluation

## Abstract

**Background:**

PTB is an infectious disease, which not only seriously affects people’s health, but also causes a heavier disease economic burden on patients. At present, reform of the medical insurance payment can be an effective method to control medical expenses. Therefore, our study is to explore the compensation mechanism for pulmonary tuberculosis (PTB) patients with a full period of treatment, to alleviate the financial burden of PTB patients and provide a reference and basis for the reform of PTB payment methods in other regions and countries.

**Methods:**

The quantitative data of PTB patients was collected from the first half of 2015 to the first half of 2018 in Dehui Tuberculosis Hospital in Jilin Province, and medical records of PTB patients registered in the first half of 2018 (*n* = 100) from the hospital was randomly selected. Descriptive analysis of these quantitative data summarized the number, cost, medication and compliance. Semi-structured in depth interviews with policymakers and physicians were conducted to understand the impact of interventions and its causes.

**Results:**

After implementation of the compensation mechanism, the number of PTB patient visits in 2018 was increased by 14.2%, average medical costs for outpatients and inpatients were significantly reduced by 31.8% and 47.0%, respectively, and the auxiliary medication costs was reduced by 36.5%. Moreover, the hospital carried out standardized management of tuberculosis, and the patient compliance was very high, reaching almost 90%.

**Conclusions:**

The capitation compensation mechanism with a full period of treatment was a suitable payment method for PTB, and it is worthy of promotion and experimentation. In addition, the model improved patient compliance and reduced the possibility of drug-resistant PTB. However, due to the short implementation time of the model in the pilot areas, the effect remains to be further observed and demonstrated.

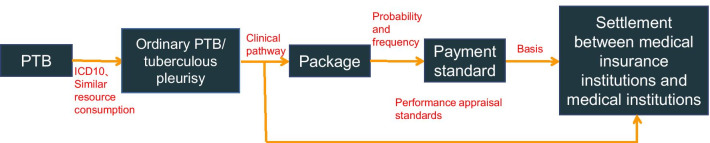

## Background

The World Health Organization “Global Tuberculosis Report 2019” pointed out that in 2018, an estimated 10 million people worldwide suffered from tuberculosis, and approximately 1.2 million people died. China is the world’s second-largest country and has a high burden of tuberculosis [1]. According to the statistics of Chinese Center for Disease Control and Prevention, the number of pulmonary tuberculosis (PTB) cases in China in 2018 was 823 342, accounting for 10.6% of the total number of infectious disease cases (ranking third in infectious diseases), and the number of PTB deaths was 3149, accounting for 13.5% of the total number of deaths from infectious diseases. PTB has become the second leading cause of death from infectious diseases, after AIDS [2]. Due to the high incidence, high mortality, and wide impact of PTB, it has become a major public health problem that seriously endangers the health of Chinese residents. PTB is also called a “poverty disease”. Eighty percent of PTB patients are concentrated in rural areas, and the economic burden of the disease is heavy. PTB has become one of the major diseases that sends individuals back into poverty due to illness, initially causing poverty due to illness and restricting rural social and economic development [3].

From an economic point of view, PTB is a contagious disease, and the spread of PTB has obvious negative external effects. If PTB is not controlled, it not only increases the medical economic cost of patients but also causes social costs that far exceed the individual costs [4]. Since health services have the special nature of supply-side leadership and demand-side passiveness, efforts to reduce the economic burden of PTB patients must start from the source to improve supplier behaviour and effectively control medical expenses. At present, reform of the medical insurance payment mechanism can be an effective method to control medical expenses.

According to the characteristics of PTB itself, based on the clinical path of diagnosis and treatment, this study formulated reasonable service packages, calculated scientific payment standards, and determined a capitation compensation mechanism among PTB patients with a full period of treatment. This change in the compensation mechanism may not only reduce the economic burden of patients’ disease but also strengthen the compliance management of PTB patients, which will further help reduce the burden on society. Additionally, this exploration of the compensation mechanism provides a reference for other regions and countries seeking the reform of payment methods.

## Methods

### Study sites

This study relied on the National Health Commission of China-Bill & Melinda Gates Foundation TB Collaboration project (hereinafter referred to as the “China-Bill & Melinda Gates project”) and selected Dehui City, Jilin Province, China, as the pilot city. The project started in 2016 and ended in 2018 and implemented a capitation compensation mechanism with a full period of treatment.

Therefore, we identified the data from January to July 2018 as the post-reform data and the data from January to July each year in 2015, 2016, and 2017 as the pre-reform data (Table 1).

**Table 1:** Implementation process of the research

Time	Content	People	Source/references
July 2016	Dehui City, Jilin Province, was identified as a pilot city		China-Bill & Melinda Gates project
November 2016	We conducted a baseline survey and collected the PTB-related policies, number of visits, medical expenses, etc	Our team	Health information system (HIS), medical record management system and medical insurance settlement system of Dehui Tuberculosis Hospital
January 2017	The clinical path of PTB was determined, include standard PTB diagnosis and treatment process	The expert group formed by Beijing Chest Hospital	"Diagnostic criteria for TB (WS288-2008)", "Guidelines for Implementing the National Tuberculosis Control Program in China (2008)", "Management Work Plan for Multidrug-resistant Tuberculosis Prevention and Control" and other documents and books
July 2017	The service package and payment standards of the hospital were determined (Table 2)	Our team	According to the established standard clinical path, combined with the actual diagnosis and treatment cost of the hospital from 2015 to 2017
August 2017	We proposed a capitation compensation mechanism with a full period of treatment	Our team	Based on the above research and argumentation
December 2017	The model was officially launched	Dehui Tuberculosis Hospital	After many discussions with relevant leaders of the hospital
July 2018	The model was over		The “China-Bill & Melinda Gates project” was completed

### Study design

This study focused on the introduction of a compensation mechanism for PTB with a full period of treatment and used a mixed-methods approach. We combine quantitative analyses of PTB case records from the Hospital Information System (HIS), medical record management system and medical insurance settlement system of Dehui Tuberculosis Hospital, and semi-structured in depth interviews with policymakers and physicians to evaluate its implementation effects.

### Data collection

We searched tuberculosis as the main diagnosis in HIS, medical record management system and medical insurance settlement system of Dehui Tuberculosis Hospital. The time was selected from the first half of 2015 to the first half of 2018, and the search results were all included in the scope of the study.

The dataset contained three basic sets of information: (1) the number of PTB patients, including the number of outpatients and inpatients and the proportion of different types of PTB patients; (2) the cost of PTB patients, including total cost of outpatients and inpatients, average cost, hospital day (HOD), etc. (3) the treatment behaviour of PTB patients, mainly including hepatoprotective drug costs and auxiliary medication costs and their respective proportions.

These records were examined by clinical experts according to the following constraints: (1) the main diagnosis is PTB; (2) the rationality of treatment regimen, defined as whether drugs in the prescription were selected based on PTB disease condition using the reasonable drug treatment by National Diagnostic and Treatment Standards.

In addition to, we conducted a sampling survey and searched all 1116 PTB patients in the first half of 2018 in the medical record management system of Dehui Tuberculosis Hospital. We randomly selected 100 patients based on the patient’s hospital number and name as the search criteria, and checked his medical records. The main observation indicators included the number and date of the patient’s medication and examination, as well as to estimate the patient’s compliance.

### Qualitative data

We conducted semi-structured in-depth interviews to understand the effects of the intervention during the project and the reasons for the problems. In-depth interviews were conducted with the staff of the provincial and prefectural health committees (*n* = 6), the Centers for Disease Control and Prevention (*n* = 3), the Health Insurance Bureau (*n* = 3) and the insurance department of the Dehui Infectious Disease Hospital (*n* = 2), to understand the operating effects, the problems and countermeasures that occurred during the operation of the mode. Conducted in-depth interviews with physicians to learn about patients’ perception and changes in patients’ medical behavior and compliance of the model.

### Data analysis

A descriptive analysis was performed on the patients evaluated in the cohort. Continuous variables were described using mean and the trend of changes over the same period. Categorical variables were summarized using numbers and percentages.

The operation effect of this mode for PTB patients was evaluated in the four areas: patients visits, patient expenses, drug types and costs, and patient compliance.

Indicators were calculated which the data from January to July each year in 2015, 2016, and 2017 as the pre-reform data, and the data from January to July 2018 as the post-reform data. Compared the cohort data over the same period to see if there is a significant difference between the baseline and the final value.

## Mode implementation background and introduction

### Mode implementation background

According to the baseline survey, Dehui Tuberculosis Hospital used a project-based payment method before the reform was implemented. In this hospital, PTB has the following characteristics: (1) High hospitalization rate. Since the medical insurance policy of Dehui City does not reimburse the outpatient expenses of tuberculosis patients, people often choose to be directly hospitalized when diagnosed with ordinary PTB, which results in a hospitalization rate of tuberculosis patients as high as 60%. (2) High medical expenses. The hospitalized patients include those with the four types of ordinary initial PTB, ordinary retreatment PTB, PTB pleurisy, and PTB with comorbidities or complications (excluding drug-resistant PTB). The average hospitalization costs for these types of PTB in 2016 were Chinese Yuan (CNY) 6608, CNY 6545, CNY 7549, and CNY 8093, respectively. Among them, the proportion of PTB cases with adverse reactions or complications was as high as 16.5%, which is one of the reasons for the high cost of PTB in this hospital. (3) Unreasonable cost structure. Through the analysis of the diagnosis and treatment items of PTB patients in the hospital in 2016, it was found that the diagnosis and treatment items of PTB included drug fees, bacteriological examination fees, imaging examination fees, routine examination fees, diagnosis and treatment service fees, and other expenses. Among them, drug fees and diagnosis and treatment service fees accounted for more than 70.0%, while diagnosis and examination expenses accounted for less than 3.0%, and routine examinations accounted for only 4.9%. Other expenses (mainly liver protection drugs) were as high as 21.3%. This shows that the hospital paid attention to the treatment of PTB but neglected PTB screening and diagnosis and the routine monitoring of the patient’s condition. (4) High out-of-pocket rate of tuberculosis patients. The baseline survey showed that the actual compensation ratio for tuberculosis patients in the hospital was 67.5%, and the patient’s out-of-pocket rate was much higher than 30.0% (required by the “China-Bill & Melinda Gates project”).

### Model design basis and principles

**Fig. 1:**
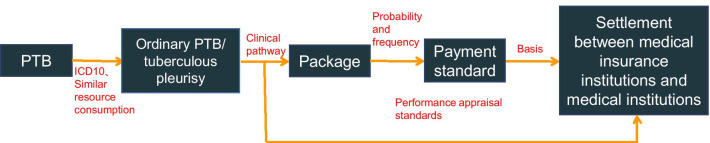
Compensation mechanism for PTB with a full period of treatment. *PTB* Pulmonary tuberculosis

1. One of the effective measures to control medical expenses is the reform of medical insurance payment methods. We drew on the idea of capitation and divided PTB into two categories: ordinary PTB and drug-resistant PTB (not involved in the study). Ordinary PTB also included ordinary initial and retreatment PTB, PTB with comorbidities or complications (calculated according to the incidence), and PTB pleurisy. The medical insurance agency presented medical service packages according to the clinical path of each type of PTB, and after reasonable calculation, determined the fixed fee payment standard for each patient (see Table 2) to be paid to the medical service provider (see Fig. 1). This represented a payment system reform suitable for chronic disease management. Its advantages included the promotion of the active control of medical service providers, the strengthening of health education for patients and the standardized management of the entire course of treatment. The disadvantage was the difficulty of determining a reasonable payment standard. Prepayments that were too high or too low had an impact on medical service providers [5, 6].
2. The disease characteristics of PTB are presented as follows. First, PTB is a disease that can be cured through standardized treatment. Second, PTB has a relatively clear diagnosis and treatment process. There are standardized treatment plans for PTB in China and around the world, which provide a reference for specific service packages. Finally, if the compliance of PTB patients is improved, the treatment effect can be very good, and the occurrence of drug-resistant PTB can be avoided. Therefore, we designed medical insurance payment methods for PTB and provided references for other diseases with relatively clear diagnosis and treatment procedures.

**Table 2:** Quota standards for different types of PTB

Single disease category code	Single disease category name	Treatment	ICD code	Norm standard (CNY)^ab^
CD22000101	Ordinary PTB	Outpatient	A15.300	3600
CD22000102	Ordinary PTB	Full period (outpatient + hospitalization)	A15.300	9000
CD22000201	PTB pleurisy	Outpatient	A16.500 × 004	5000
CD22000202	PTB pleurisy	Full period (outpatient + hospitalization)	A16.500 × 004	12 000

*PTB* Pulmonary tuberculosis, *CNY* Chinese Yuan

^a^The standard for the full period of treatment was calculated based on the probability of adverse reactions and complications of PTB

^b^The cost standard is the fixed standard given by medical insurance institutions to medical institutions, and it does not include free first-line anti-tuberculosis drugs invested in by the state or government

### Model introduction

To alleviate the economic burden of PTB patients and solve the problem of a high hospitalization rate, Dehui Tuberculosis Hospital reformed the medical insurance payment method and designed a capitation compensation mechanism with a full period of treatment. For the policy changes before and after implementation of the reform, refer to Table 3.

**Table 3:** Policy changes before and after the implementation of the model

Index	Before	After
PTB classification	No	Ordinary PTB/PTB with comorbidities and complications/PTB pleurisy
Medical insurance payment	Fee-for-services	Compensation mechanism with a full period of treatment
Compensation plan		
Outpatient	Special diseases, according to hospitalization compensation	Outpatient (according to the norm standard, Table 2)
Inpatient	Deductible is 600 RMB, The reimbursement rates for county, township, and village-level medical institutions are 50%, 55%, and 75%, respectively Medical institutions outside the county are not reimbursed	Full period (according to the norm standard, Table 2) Cancel the deductible and the non-reimbursable project expenses outside the medical insurance catalogue 70% reimbursement according to the norm standard Cancellation and project expenses and deductible health insurance directory can not be reimbursed
Billing plan		
Medical institutions and medical insurance institutions	Hospital compensation amount = reimbursable expenses × reimbursement ratio Reimbursable expenses = total medical expenses − non-reimbursable expenses − deductible line	Hospital compensation amount = fixed payment standard × reimbursement ratio (70%) × number of patients
Medical institutions and patients	Patient out-of-pocket expenses = total medical expenses − hospital compensation expenses	Patient out-of-pocket expenses = total medical expenses × out-of-pocket ratio (30%)
Assessment standard	No	Whether to implement according to the clinical path, if yes, all funds are allocated according to the service package payment standard; if not, 10% of the funds are deducted

*PTB* Pulmonary tuberculosis

### Model features

The model did not address the reimbursement of hospitalization expenses alone or merely increase the expense reimbursement ratio but rather involved a medical insurance fund payment implementation plan that combined outpatient care and hospitalization. Based on the characteristics of tuberculosis disease, the plan enhanced the importance of prevention based on the principles of prevention first and standardized management to change the medical insurance payment model from the original post-payment model to the pre-payment model. The method of standardized treatment was adopted according to the clinical path, and the surplus funds were returned to the individual, which changed the hospital’s operation model from being treatment-oriented to being prevention-oriented.

## Results

### Changes in the number of visits for PTB

Dehui City Tuberculosis Hospital treated TB, including PTB, bone TB, renal TB, TB meningitis and other TB, but PTB was the main treatment disease, accounting for 90% of patients in the whole hospital. As noted above, the hospital implemented the compensation mechanism with a full period of treatment in December 2017.

From 2015 to 2017, the number of patient visits to the hospital decreased year by year, but after the implementation of the payment method reform in 2017, the number of visits of PTB patients in 2018 increased by 14.2%, and both outpatients and inpatients increased. Compared with the decrease of 2.9% in the new incidence of PTB in the region (Jilin Province) in 2018, it can be seen that the implementation of the payment method reform played a certain role in the recovery of the number of PTB patients in the hospital (Table 4).

**Table 4:** Changes in the number of hospital visits for PTB in the first half of 2015–2018

Year	Regional incidence (1/100 000)	Number of visits for PTB					
		Total	Growth %	Inpatient	Growth %	Outpatient	Growth %
First half of 2015	56.5	1432	-	255	-	1177	-
First half of 2016	49.8	1304	−8.9	165	−35.3	1139	−3.2
First half of 2017	50.3	977	−25.1	68	−58.8	909	−20.2
First half of 2018	47.4	1116	14.2	78	14.7	1038	14.2

### Changes in the average costs for PTB

Average medical costs are an important indicator that reflects the situation of medical expenses. After the hospital implemented a full period capitation compensation mechanism at the end of 2017, the average outpatient cost in 2018 was reduced by 31.8% to almost half of the average cost in 2017, and the average inpatient cost decreased by 47.0% year-on-year. The average cost of outpatient and inpatient visits was greatly controlled. This shows that the model had a significant effect on cost control (Table 5).

**Table 5:** Changes in the average costs for PTB in the hospital in the first half of 2015–2018 (Unit: CNY)

Year	Inpatient	Growth	Outpatient	Growth
First half of 2015	527	-	7364	-
First half of 2016	527	0.0%	6437	−12.6%
First half of 2017	523	−0.8%	6084	−5.5%
First half of 2018	277	−47.0%	4152	−31.8%

*CNY* Chinese Yuan, *PTB* Pulmonary tuberculosis

### Changes in PTB treatment behaviour

Drug therapy is a major method adopted in the clinical treatment of PTB. During the treatment of PTB patients from 2015 to 2017, the cost of adjuvant drugs accounted for a large part of the total medical cost, while the cost of liver-protecting drugs accounted for only approximately 13%. After the implementation of the capitation compensation mechanism with a full period in 2017, the cost of adjuvant drugs for the treatment of PTB was greatly reduced in 2018, and the cost of liver protection drugs increased. This model limited the abuse of auxiliary drugs in the hospital. Further analysis shows that one of the reasons for the substantial reduction in the cost of adjuvant medication was the reduction in the types of adjuvant medications, from an average of 2.8 in 2015 to 1.4 in 2018. After the reform, the hospital reduced the use of non-essential auxiliary drugs, and standardized treatment behaviour improved (Table 6).

**Table 6:** Changes in the cost of hepatoprotective drugs and auxiliary drugs in the hospital in the first half of 2015–2018 (Unit: CNY)

Year	Total medical costs	Growth %	Hepatoprotective drug costs (yuan)	Growth %	Percentage	Auxiliary medication costs	Growth %	Percentage
First half of 2015	4897	-	654	-	13.4	1699	-	34.7
First half of 2016	5124	4.6	676	3.3	13.2	1752	3.1	34.2
First half of 2017	4599	−10.2	606	−10.3	13.2	2091	19.3	45.5
First half of 2018	4333	−5.8	656	8.2	15.1	1329	−36.5	30.7

*CNY* Chinese Yuan

To address whether the hospital presented sufficient diagnosis and treatment to reduce medical expenses, we researched the average length of hospitalization of PTB patients. The results showed that the average length of hospitalization for PTB patients increased year by year, from 20 days in 2015 to 25 days in 2018. The standard length of hospitalization for the clinical pathway of ordinary PTB was 25 days, and the average length of hospitalization after the reform in 2018 was close to the standard value.

We interviewed hospital administrators and learned that after the reform, when PTB patients were admitted to the hospital, medical staff performed diagnosis and treatment according to the type of PTB and basically followed the service package items set up in accordance with the clinical pathway specifications, so the average length of stay was close to the standard situation. However, we did not further analyse and verify the diagnosis and treatment items of each patient, so this conclusion is for reference only.

### Changes in PTB patient compliance

Compliance can also be called compliance behaviour, which refers to the consistency of a patient’s diet, medication, lifestyle, etc. with his or her doctor’s advice. It can be divided into three categories: complete compliance, in which the patient shows standardized compliance with his or her doctor’s advice and executes it as scheduled; partial compliance, in which compliance cannot be effectively completed for some reason and there are cases in which execution of the doctor’s orders is omitted; and non-compliance, in which the changes are made to the doctor’s orders without authorization or the patient does not at all follow his or her doctor's advice, buys drugs, stops taking drugs, or abuses drugs [7]. PTB is a disease that can be cured through regular medication. The patient’s treatment compliance will directly affect the effect of treatment and the progress of the disease. Therefore, in the process of treating PTB patients, the importance of treatment compliance should be strengthened [8].

We randomly selected 100 patient cases for review in the first half of 2018, and the results showed that patient compliance was very high, reaching almost 90%. Through further interviews with doctors, we found that the previous management model was changed after the payment reform. Not only were diagnosis and treatment conducted in the hospital according to standardized procedures, but discharged patients received follow-up calls by phone, with attention to drug consumption and regular review after discharge to promptly identify patients who stopped taking medication or did not undergo regular review. Then, the hospital contacted patients through the TB prevention network and directed them to the TB prevention institution for treatment. Therefore, the vast majority of patients took their medication and regularly attended their check-up appointments (Table 7).

**Table 7:** Compliance of hospital pulmonary tuberculosis patients

Compliance	Take medicine	Proportion	Check	Proportion
Complete compliance^a^	62	62%	23	23%
Partial compliance^b^	35	35%	62	62%
Non-compliance^c^	3	3%	15	15%
Total	100	1	100	1

^a^Complete compliance: Refers to taking medication and checking as prescribed by a doctor for 6 consecutive months and above

^b^Partial compliance: Refers to taking medication and checking as prescribed by a doctor for 1–6 months in a row

^c^Non-compliance: Refers to not taking medication and checking as prescribed by the doctor

## Discussion

### The capitation compensation mechanism with a full period is suitable for PTB disease and is worthy of promotion and experimentation

After years of clinical practice and exploration, China has developed clear technical specifications for the prevention and treatment of PTB. If the patient can be treated in accordance with standardized procedures, the disease can essentially be cured. However, the current medical insurance reimbursement method for PTB covers mainly hospitalization, which leads to the problem of a high tuberculosis hospitalization rate and increases medical insurance fund expenditures. We adopted the capitation compensation mechanism with a full period and combined outpatient treatment and inpatient treatment to promote the effective control of tuberculosis patients in the outpatient stage, prevent patients from undergoing additional hospitalization to obtain medical insurance reimbursement, and use medical insurance funds more efficiently. Additionally, the surplus was used to encourage medical institutions to carry out PTB management and to improve patients’ compliance with standardized treatment.

### The medical expenses of TB patients have decreased significantly, and the economic burden of the disease has been reduced

The average cost is an indicator that directly reflects the cost control effect of the payment method. As medical costs increase yearly with economic growth, the change in average cost is also a direct indicator to evaluate the effect of medical cost control. Compared with the situation before the implementation of the project, the average outpatient cost for the hospital decreased by 47.0%, the average inpatient cost decreased by 39.0%, and the average costs of outpatient services and hospitalization were greatly reduced. The payment method reform had a significant effect on cost control. Moreover, the reimbursement ratio of the new plan was 70%, and the patient actually paid only 30% of all medical expenses, which effectively reduced the patient’s economic burden of disease.

### Clinical treatment methods have been effectively improved, the rational use of drugs has changed significantly, and the standardized management of diagnosis and treatment behaviour has been improved

In the past, the drug cost of PTB patients accounted for more than 30% of the medical expenses, the use of hepatoprotective drugs averaged 2–3 species, and there were more than three kinds. The new plan required the standardized management of PTB, especially for the use of non-essential hepatoprotective drugs and auxiliary drugs. After the implementation of the capitation compensation mechanism with a full period of treatment, the cost of auxiliary drugs was greatly reduced, and the types of liver protection drugs gradually decreased and stabilized. The effect of rational drug use began to appear. Additionally, the hospital began to pay attention to and strengthen the awareness of the management with a full period of treatment, minimize hospitalization, reduce costs, and improve the standardized management of PTB.

### Patient compliance is improved, which reduces the possibility of the development of drug-resistant PTB

This model effectively improved patient compliance through clinical path management inside the hospital and tracking management outside the hospital. Patients with drug-resistant PTB have two routes of infection. The first route is the development of common tuberculosis. Due to the poor compliance of the patient, treatment is not performed as prescribed by the doctor, which leads to bacterial resistance to PTB drugs. The second route involves infection with drug-resistant strains. The source of infection is drug-resistant patients; thus, the first infection of the patient is drug resistant. Therefore, improving patient compliance is beneficial for reducing the possibility of drug resistance in patients [9]. Because tuberculosis is contagious and has obvious negative external effects, the model that reduced the incidence of tuberculosis and drug-resistant tuberculosis also reduced the burden on society as a whole.

The deficiencies of this research point out that there are two aspects: First, the model selected Dehui Infectious Disease Hospital as a pilot operation for half a year, and the initial effect has been shown. However, due to the short implementation period and single pilot, further long-term promotion in other regions will be needed in the future to verify the effect of this model. Second, the statistical analysis of this study is relatively simple, mainly descriptive analysis without using the model for precise analysis. The above content is also our future research direction.

## Conclusions

This model is worthy of promotion. We used a hospital as a pilot and adopted the capitation compensation mechanism with a full period of treatment. The core idea of this model is to combine prevention and treatment, striving to prevent patients from becoming ill and to keep illnesses minor. This method can improve the standardized treatment and reduce the infection rate of PTB patients, thereby reducing the impact of PTB on society. The initial operational effect of this model is satisfactory, but the implementation period is short, and further follow-up observation is needed. Future studies may discuss insufficient diagnosis and treatment services and further refine this model, which may also provide a reference for the reform of PTB payment methods in other regions or countries.

## Data Availability

All data generated or analysed during this study were kept confidential by Dehui Tuberculosis Hospital in Jilin Province, China. The datasets are available from the corresponding author on reasonable request.

## Abbreviations

TB: Tuberculosis
PTB: Pulmonary tuberculosis
CNY: Chinese Yuan
AIDS: Acquired immune deficiency syndrome
HIS: Health information system
WS288-2008: Diagnostic criteria for pulmonary tuberculosis (WS288-2008)
HOD: Hospital day
